# The role of implicit social bias on holistic processing of out-group faces

**DOI:** 10.1186/s41235-023-00464-3

**Published:** 2023-01-26

**Authors:** Wei Chen, Mahlet T. Kassa, Olivia S. Cheung

**Affiliations:** 1 Department of Psychology, Science Division, New York University Abu Dhabi, Abu Dhabi, UAE; 2 College of Arts and Sciences, Khalifa University of Science and Technology, Abu Dhabi, UAE; 3 Berlin School of Mind and Brain, Humboldt Universität Zu Berlin, Berlin, Germany

**Keywords:** Face perception, Implicit association test, Composite task, Individual differences

## Abstract

Although faces of in-group members are generally thought to be processed holistically, there are mixed findings on whether holistic processing remains robust for faces of out-group members and what factors contribute to holistic processing of out-group faces. This study examined how implicit social bias, experience with out-group members, and ability to process in-group faces holistically might predict the magnitude of holistic processing for faces of two out-groups: other-race and other-age groups. In Experiment 1, Caucasian participants viewed Caucasian (own-race) and East Asian (other-race) faces. In Experiment 2, young adult participants viewed young adult (own-age) and baby (other-age) faces. Each participant completed a composite task with in-group and out-group faces, an implicit association test, and questionnaires about their experience with in-group and out-group members. We found that while the participants had relatively extensive experience with the other-race group, they had limited experience with the other-age group. Nonetheless, implicit social bias was found to positively predict the magnitude of holistic processing for both other-race and other-age faces. Exploratory analyses on the interactions among the predictors suggest that the effect of implicit social bias was primarily observed in participants with strong holistic processing ability of in-group faces but with low level of experience with members of the out-groups. These findings suggest that observers utilize different kinds of information when processing out-group faces, and that social features, such as race or age, are incorporated to influence how out-group faces are processed efficiently.

## Significance statement

Face recognition is critical for everyday social interactions. At a glance of a face, different aspects of information, including identity (e.g., Jennifer Lawrence), race, age, or emotion, may be extracted. Although it is possible to rely on salient visual features, such as skin tone or forehead–chin proportion for race or age categorization, successful recognition of individual faces often relies on holistic processing—the ability to process all facial features as a whole—to differentiate individual faces. It has been shown that recognition of out-group faces, such as faces from a different race or age group, is often worse than recognition of in-group faces, with mixed findings on whether holistic processing is also less engaged for out-group than in-group faces. This study examined how holistic processing of two different out-groups (Caucasian participants viewing East Asian faces, or young adult participants viewing baby faces) may be predicted by three factors: implicit social bias toward the out-group, social contact experience with members from the out-group, and holistic processing ability for in-group faces. Across two experiments, we found that positive implicit social biases toward the out-groups (East Asians or babies) led to stronger holistic processing for those faces. This effect was found to be strongest in observers who had strong holistic processing for in-group faces and little experience with the out-group. These findings suggest that holistic processing of faces from an out-group depends on multiple factors including implicit social bias, but substantial experience with members of the out-groups could potentially eliminate such socio-cognitive influences.

## Introduction

Successful categorization and recognition of faces is critical for our everyday social interactions. Most people can recognize a large number of individual faces, and extract multiple aspects of useful information for social interactions, such as the other person’s identity, race, age, gender, emotions, or perceived personality traits including trustworthiness. Although it is possible to rely on salient visual features to easily categorize faces into various race, age, or gender groups, it often requires the processing of all facial features and their spatial configurations to discriminate and identify individual faces. Indeed, faces are thought to be processed more holistically than other objects, with important facial features processed in an integrative manner (Tanaka & Farah, 1993; Young et al., 1987). Most people are proficient in processing faces that are from their familiar groups (e.g., own-race or own-age groups). In increasingly globalized societies, it is also important to be able to efficiently process faces and identify individuals across various demographics to facilitate social exchanges. However, it remains unclear whether and how out-group faces are processed efficiently in a holistic manner.

This study aimed to examine the factors that contribute to the holistic processing of faces from two well-studied out-groups: the other-race and other-age groups. The other-race effect reveals that people recognize faces from their own race more accurately than those from other races (Malpass & Kravitz, 1969; Anthony et al., 1992; Meissner & Brigham, 2001). Similarly, the other-age effect suggests that recognition is better for faces of the participants’ own-age group than those of either older or younger age groups (e.g., elderly or children for young adult participants, Rhodes & Anastasi, 2012).

Because holistic processing is thought to be critical for face recognition, earlier findings suggested that holistic processing is also more robust for own-race or own-age faces, compared with other-race or other-age faces (e.g., other-race: Michel et al., 2006a, 2006b; DeGutis et al., 2013; other-age: de Heering & Rossion, 2008; see also Wiese et al., 2013). For instance, Caucasian participants showed better performance in recognizing whole faces than isolated face parts (e.g., eyes) for Caucasian faces, but the difference in recognition performance between wholes vs. parts was reduced for Asian faces (Michel et al., 2006a; Tanaka et al., 2004). Likewise, children showed stronger holistic processing than adults for child faces (Susilo et al., 2009) and adults who had limited experience with young children also showed stronger holistic processing for adult than child faces (de Heering & Rossion, 2008), suggesting that own-age faces may also be processed more holistically than other-age faces. Apart from group memberships as indicated by visual differences between race or age groups, several studies suggested that the magnitudes of holistic processing was also different for faces that were arbitrarily assigned as members of in-group vs. out-group (e.g., young adult faces randomly assigned to own- vs. other-university affiliations: Hugenberg & Corneille, 2009; or perceptual adaption of racially ambiguous faces as own- vs. other-race faces: Michel et al., 2010).

However, recent studies showed comparable holistic effects for both own- and other-race/age faces (e.g., Harrison et al., 2014; Horry et al., 2015; Wong et al., 2021). For instance, at the group level, comparable holistic processing for own-race and other-race faces for Caucasian, Asian, or Black participants has been observed (Bukach et al., 2012; Harrison et al., 2014; Mondloch et al., 2010; Zhao et al., 2014). Moreover, comparable magnitudes of holistic processing were also found for faces that were arbitrarily assigned as in- vs. out-groups (Harrison et al., 2014). In addition to the comparable holistic effects for in-group and out-group faces, studies on individual differences also suggest similar holistic processing may be engaged for faces from different groups, as the magnitudes of holistic processing for own-race and other-race faces were significantly correlated (Horry et al., 2015).

The mixed findings in the literature could potentially be due to the fact that face recognition is a complex cognitive process which involves various factors. In particular, experience and socio-cognitive factors are two major factors that have been suggested to affect the magnitude of holistic processing of out-group faces and the differences in the magnitude of holistic processing for in-group and out-group faces. Indeed, the amount and quality of experience with the out-group appear to be critical. Specifically, extensive experience in identifying individuals, instead of merely categorizing individuals into groups, appears to be essential to improve holistic processing for out-group faces (Bukach et al., 2012; Walker & Hewstone, 2006; see also Zhao et al., 2014). For example, Caucasian and Black participants who had more experience with individuals from the other race showed stronger holistic effects than those with little experience (Bukach et al., 2012). Similarly, preschool teachers who had extensive experience with young children also showed increased holistic processing for child faces than other adults who rarely interacted with young children (de Heering & Rossion, 2008).

Apart from the role of experience, the processing of out-group faces is also thought to be influenced by socio-cognitive factors. Indeed, out-group faces may be first categorized according to their group membership instead of their individual identities (Hugenberg et al., 2007; Levin, 2000). Moreover, it has been suggested that the lack of motivation to individuate out-group faces, instead of the lack of the experience to individuate faces of out-group members, leads to reduced use of holistic processing for the out-group faces (Hugenberg & Corneille, 2009; Hugenberg et al., 2007; see Hugenberg et al., 2010 for a review). Nonetheless, several studies found that socio-motivational influences might not be readily observed in all situations and could be context-specific (Kloth et al., 2014; Tullis et al., 2014; Wan et al., 2015) and that comparable holistic processing for in-group and out-group faces has been observed despite socio-motivational instructions (Harrison et al., 2014; Horry et al., 2015; Zhao et al., 2014).

To further elucidate the multiple sources of influences on the processing of out-group faces, the current study focused on individual differences to examine the effects of experience and socio-cognitive factors on the holistic processing of such faces. Instead of highlighting group membership in previous studies on holistic processing, here we examined how implicit social biases might affect holistic processing of other-race faces (Experiment 1) and other-age faces (Experiment 2). Note that although the influence of implicit social biases on face recognition performance have been examined (Lebrecht et al., 2009; Trawiński et al., 2021; Walker & Hewstone, 2008), it remains unclear how it might influence holistic processing—a hallmark of processing of own-group faces. It is possible that positive implicit biases toward other race or age groups could enhance holistic processing of those out-group faces, because previous findings have shown that positive emotions lead to increased holistic face processing (Chen & Cheung, 2021; Curby et al., 2012; Xie & Zhang, 2016).

In addition to the potential influences of implicit social bias toward and experience with the out-group members, it is also important to note that the magnitude of holistic processing vary substantially across participants (DeGutis et al., 2013; Richler et al., 2011; Wang et al., 2012; Horry et al., 2015; see also Konar et al., 2010), and that significant correlations have been observed between the magnitudes of holistic processing of in-group and out-group faces (Horry et al., 2015). Therefore, we also took into account the magnitude of individual participants’ holistic processing for in-group faces to predict the magnitude of holistic processing for out-group faces across participants.

In Experiment 1, Caucasian participants were shown Caucasian (own-race) and East Asian (other-race) faces. In Experiment 2, young adult participants were shown young adult faces (own-age) and baby (other-age) faces. The use of East Asian and baby faces was due to the potential wide range of positive to negative implicit social biases toward these groups (e.g., Greenwald et al., 1998; Senese et al., 2013), because unfortunately a relatively narrow range of implicit social biases, specifically negative implicit social biases or stereotypes, toward certain groups such as Black or elderly people have been consistently observed (Charlesworth & Banaji, 2022). Although a relatively wide range of positive and negative implicit social biases was expected for East Asians from Caucasians and for babies from young adults, we expected that participants’ experience with these out-groups might differ. Specifically, our participants were university students living on a highly culturally diverse campus in an international city and likely had relatively high experience with individuals from different races (e.g., Caucasian and East Asian). In contrast, the participants likely had relatively little experience with babies.

Apart from reporting the group-level results for transparency and completeness, the main hypotheses of the current study focused on individual differences. Both Experiments 1 and 2 had three predictors, holistic processing of in-group faces, experience with individuals of the out-group, and implicit social bias toward the out-group. For the three predictors, we first expected that the magnitude of holistic processing for in-group faces would positively predict the magnitude of holistic processing for out-group faces. Second, experience with individuals of the out-group should also positively predict the magnitude of holistic processing of out-group faces. Third, holistic processing of out-group faces might also be predicted by implicit social bias, with a strong positive implicit bias toward an out-group predicting an increase in holistic processing for out-group faces. Moreover, we explored the potential interactions among these predictors, because the influence of a predictor might depend on the level of another predictor.

In both experiments, holistic processing was indexed by the congruency effect in a face composite task, similar to the complete design of the composite task (Richler et al., 2011, 2012). Following the Vanderbilt Holistic Processing Task which aims to measure individual differences (Richler et al., 2014; Wang et al., 2016), only aligned, but not misaligned, composites were included in this study, because aligned trials accounted for most of the variance in the task (Horry et al., 2015; Richler & Gauthier, 2014; Wang et al., 2016). Holistic processing was revealed by better or faster performance for congruent than incongruent trials (e.g., Cheung & Gauthier, 2010; Curby et al., 2012; Richler et al., 2011).

## Experiment 1

### Method

#### Participants

A total of 57 Caucasian undergraduate students (41 females and 16 males, mean age = 19.5 years, SD = 1.42) at New York University Abu Dhabi completed Experiment 1 for course credits or subsistence allowance. The university has a highly diverse student population (approximately 2000 undergraduate from over 85 countries; most students live on campus). All participants reported normal or corrected-to-normal vision and provided written informed consent approved by the New York University Abu Dhabi Institutional Review Board. The sample size of a minimum number of 50 participants was chosen to be comparable or larger than those used in previous studies, such as Walker and Hewstone (2006, 2008). Three participants were excluded due to low performance in the face composite task (mean accuracy below 65%), and thus, the final sample consisted of data from 54 participants.

#### Stimuli

##### Face composite task

**Fig. 1:**
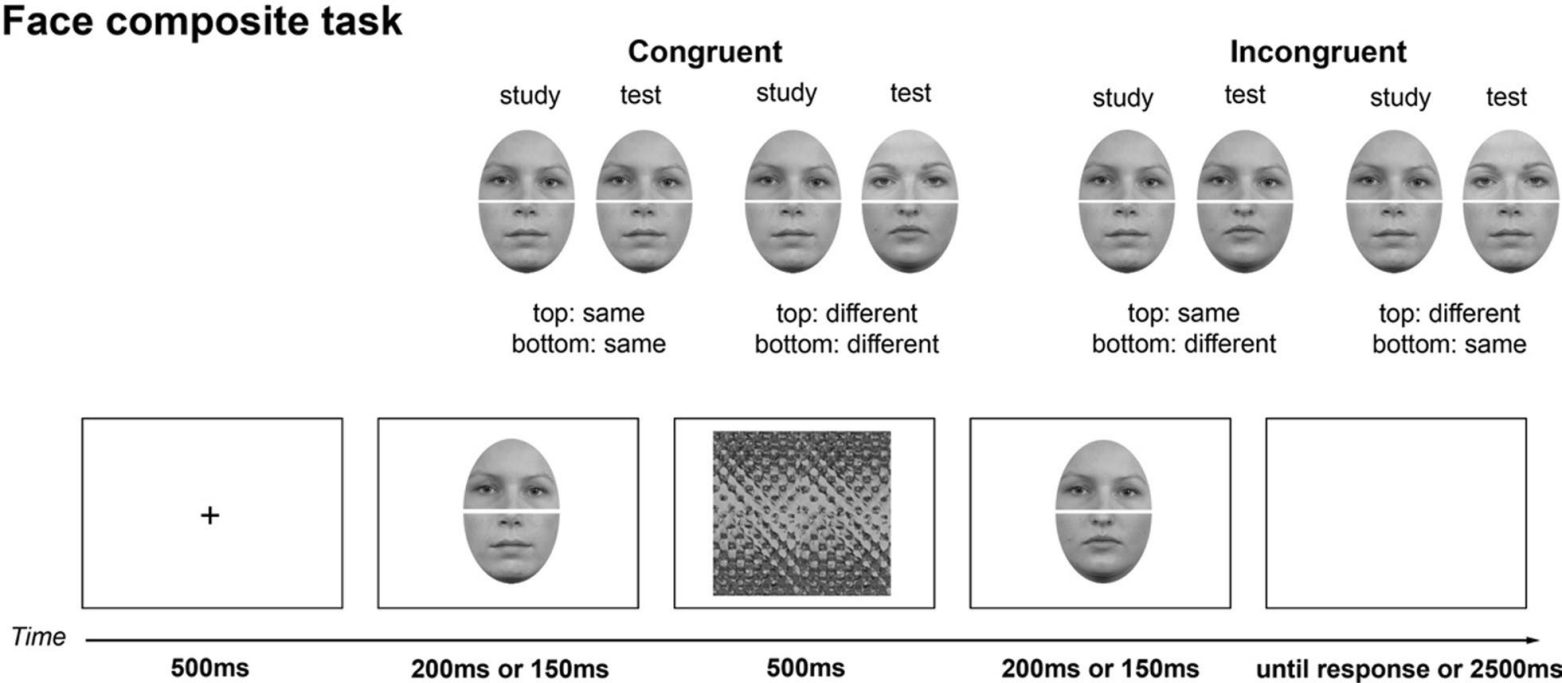
Task design and sample trial sequence of the face composite task (not actual stimuli used in the experiment due to copyright issues; the sample faces were taken from the Radboud Faces Database, Langner et al., 2010). In this task, the face composites were always aligned. The relationship between the top and bottom halves of the study and test faces could be either congruent or incongruent. To improve reliability of the measure by increasing variability in task difficulty (Wang et al., 2016), the faces were shown for 200 ms in the first half of the study and for 150 ms in the second half of the study

Figure 1 illustrates sample Caucasian stimuli in this task. The composite task included a total of 10 Caucasian female from the FACES database (Ebner et al., 2010) and 10 East Asian female face images from the University of Hong Kong Face database run by William Hayward. Only five top and five bottom face halves out of the 10 faces from each race were used. We used a small number of faces because it could improve the reliability of the composite effect (Ross et al., 2015). All faces were in frontal view. An oval was placed over each face to exclude external features (e.g., hair). The five top and five bottom face halves of the same race were paired randomly to form composites during the experiment. The top and bottom halves of the study and test faces on each trial were always aligned, similar to the design used in the previous studies that examined individual differences in holistic processing (Richler et al., 2014; Wang et al., 2016). The height and width of the face composites subtended 6° and 4.1°. On each trial, the relationship between the identities of the top and bottom halves of a study face and a test face was either congruent (i.e., top and bottom halves of the study and test faces were either both identical or both different), or incongruent (i.e., the study and test faces shared identical top halves but had different bottom halves, or vice versa).

##### Implicit association test (IAT)

In this task, a total of six Caucasian faces and six East Asian faces (3 female and 3 male for each race) from the same databases as in the composite task and a total of six positive words (savior, kindness, pleasure, happy, friend, honest) and six negative words (hatred, traitor, terrible, brutal, abuse, useless) were used. The face stimuli were modified using the same procedure as in the composite task. The words were chosen as they were comparable in word length (average length: 6.5 letters) and word frequency (average Log_10_ frequency was 1.73 for positive words and 1.15 for negative words, *t*_10_ = − 1.50, *p* = 0.16), according to the SUBTLEX-US database (Brysbaert & New, 2009), but were significantly different in ratings for positive vs. negative meanings (6.45 out of 7 for positive words, and 1.41 out of 7 for negative words, *t*_10_ = − 53.48, *p* < 0.001), according to Bellezza et al. (1986).

##### Experience questionnaire

The questionnaires were based on Walker and Hewstone (2006)’s individuating experience scale, including items regarding helping or receiving help from individuals of the different races. The items were ‘I have looked after or helped a White (an East Asian) friend when someone was causing them trouble or being mean to them,’ ‘A White (An East Asian) person has looked after me or helped me when someone was causing me trouble or being mean to me,’ ‘I have comforted a White (an East Asian) friend when they were feeling sad,’ ‘A White (An East Asian) person has comforted me when I have been feeling sad,’ and ‘I have asked a White (an East Asian) person to be on my team or in my group during sports or activities.’ The frequency was measured using a 5-point scale, with 1 indicating *never*, and 5 indicating *very often*.

#### Procedure

All participants completed three tasks in the same order: the face composite task, the implicit association test (IAT), and two questionnaires on experience interacting with either Caucasian people or East Asian people. The experiment was run on a computer using MATLAB and Psychtoolbox-3 (Brainard, 1997; Kleiner et al., 2007; Pelli, 1997).

##### Face composite task

On each trial, a fixation was presented at the center of screen for 500 ms. A study composite was then presented, followed by a pattern mask for 500 ms, then a test composite. To increase the variability of task difficulty in attempt to improve reliability for examining individual differences, the study composite and test composite were presented for 200 ms for the first half of the experiment (160 trials) and 150 ms for the second half (160 trials) of the experiment, following the procedure in Wang et al. (2016). Participants were asked to make a same/different judgment about the identity of the top halves of the face composites by pressing either the ‘s’ or ‘d’ key as accurately and quickly as possible (e.g., Cheung & Gauthier, 2010; DeGutis et al., 2013; Horry et al., 2015; Richler et al., 2011). There were a total of 320 trials, with 40 trials for each combination of face type (Caucasian/East Asian), congruency (congruent/incongruent), and correct response (same/different). All three factors were randomized.

In the current study, holistic processing was indexed by the congruency effect, indicating better and faster performance for congruent than incongruent trials (e.g., Boutet & Meinhardt-Injac, 2021; Cheung & Gauthier, 2010; Curby et al., 2012). The congruency effects can be measured in both accuracy and response times (RT). As some research had primarily reported accuracy results (Richler & Gauthier, 2014; Wang et al., 2016), other studies also reported RT results (e.g., Cheung et al., 2008; Richler et al., 2011). In this study, we reported results of both accuracy in the congruent condition minus accuracy in the incongruent condition (∆accuracy) and RT in the incongruent condition minus RT in the congruent condition (∆RT).

The reliability of the congruent trials and the incongruent trials was high in this study for own-race and other-race faces in both accuracy (*Guttman’s λ*_2_’s > 0.77) and RT (*Guttman’s* *λ*_2_’s > 0.96) in all conditions. The correlations between the congruent and incongruent trials were also high for accuracy (*r*’s > 0.498) and in RT (*r*’s > 0.957). The reliability of the difference scores between congruent and incongruent trials was calculated using the formula below (Rogosa et al., 1982; see Ross et al., 2015).

$$\rho \left(D\right)= \frac{{{\sigma }_{x1}}^{2}\rho \left({X}_{1}\right)+{{\sigma }_{x2}}^{2}\rho \left({X}_{2}\right)-2{\sigma }_{x1}{\sigma }_{x2}{\rho }_{x1x2}}{{{\sigma }_{x1}}^{2}+ {{\sigma }_{x2}}^{2}-2{\sigma }_{x1}{\sigma }_{x2}{\rho }_{x1x2}}$$

where ${\sigma }_{x1}$ is the standard deviation of the congruent trials, ${\sigma }_{x2}$ is the standard deviation of the incongruent trials, and ${\rho }_{x1x2}$ is the correlation between the two conditions. The reliability of the difference scores between congruent and incongruent trials for own-race faces were 0.490 (∆accuracy) and 0.304 (∆RT) and that for other-race faces was 0.507 (∆accuracy) and 0.258 (∆RT).

##### IAT

The race IAT followed the procedure in Greenwald et al., (1998, 2003), which involved categorization of faces as either Caucasian vs. East Asian, and words with either positive vs. negative meanings using two response keys. The relative response times in categorizing positive vs. negative attributes and Caucasian vs. East Asian faces with specific pairings of the response keys were measured.

**Fig. 2:**
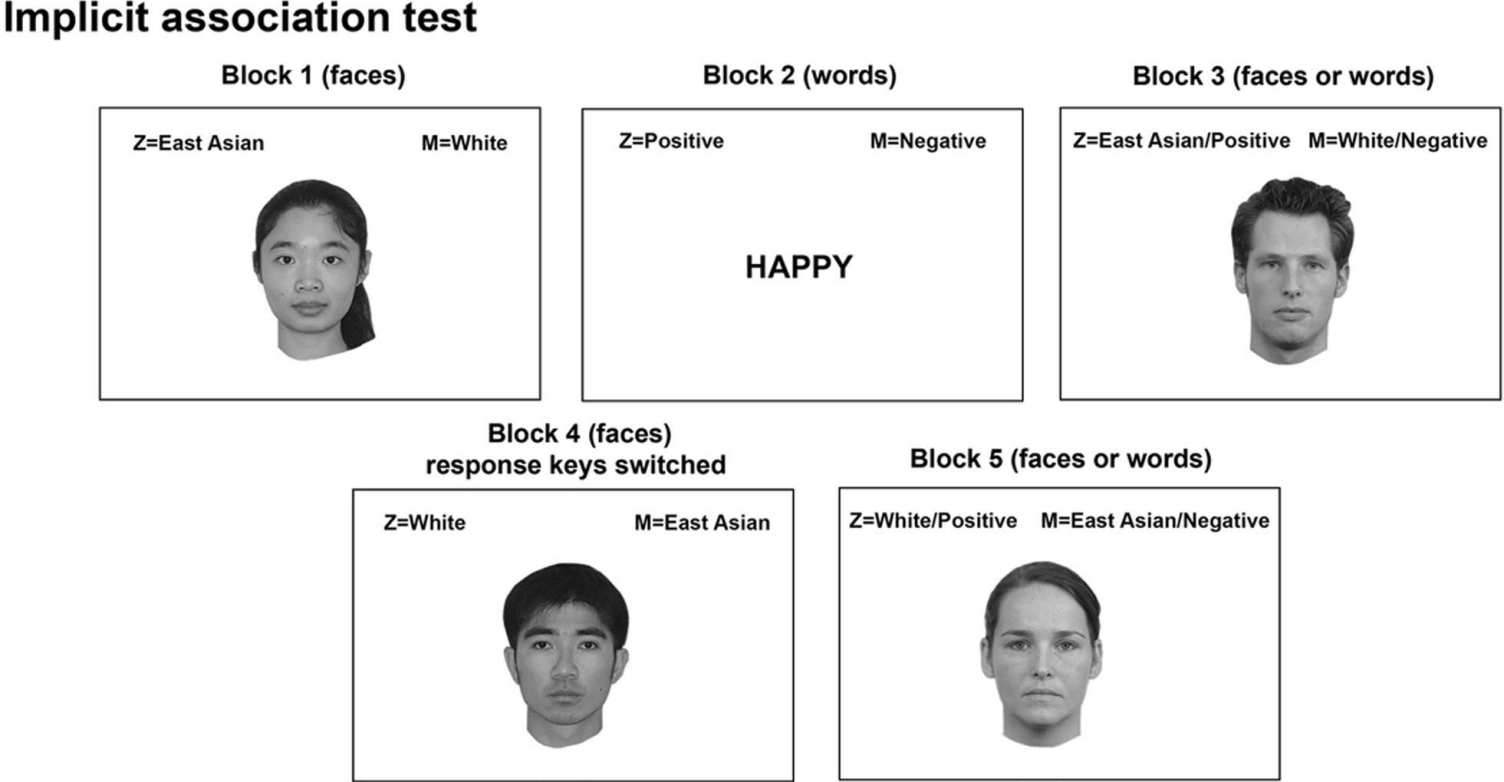
Schematic procedure of the implicit association test (IAT). Participants categorized East Asian and White faces in Block 1, and positive and negative words in Block 2. In Block 3, participants categorized the faces and words presented in a random order. In Block 4, participants again categorized the faces with the response key mapping switched. In Block 5, participants again categorized the randomly presented faces and words

The IAT involved a total of 5 blocks of trials (Fig. 2). In Block 1, participants categorized each of the presented faces as either a Caucasian or East Asian face. In Block 2, participants categorized each of the presented word as either a positive or negative word. In Block 3, face and word trials were intermixed. For half of the participants, throughout Blocks 1–3, the response key mapping was that Caucasian faces and positive words were assigned the same response key, and East Asian faces and negative words were assigned the other response key. The other half of the participants had the opposite response key mapping, with the same response key used for East Asian faces and positive words, and the other response key used for Caucasian faces and negative words. Critically, for all participants, the response key assignment for the face categories was switched after Block 3. Participants only categorized faces in Block 4, and then categorized both faces and words in Block 5, using the new response key assignment. Participants were instructed to respond as quickly and accurately as possible. A correct response was required before the next trial would begin (Greenwald et al., 2003). Blocks 1, 2, and 4 each had 40 trials. Blocks 3 and 5 each had 120 trials. Within Blocks 3 and 5, the first 40 trials were considered ‘practice’ trials and the following 80 trials were considered ‘actual’ trials (Greenwald et al., 2003). Nonetheless, all trials in Blocks 3 and 5 were used to calculate the final D scores, following the scoring algorithm suggested by Greenwald et al. (2003). Note that for the trials that participants made an initial error, response times were calculated from the onset of the stimuli to the correct responses. A positive D score indicated positive implicit bias toward East Asians and a negative D score indicated negative implicit bias toward East Asians.

For the reliability of the race IAT in the current study, Cronbach’s $\alpha$ was 0.652 and the correlations between the practice versus test trials were *r* = 0.72, *p* < 0.0001 in the Caucasian-positive/Asian-negative block, and *r* = 0.64, *p* < 0.001 in the Caucasian-negative/Asian-positive block.

##### Experience questionnaire

Participants reported the frequency of interactions with Caucasian and East Asian individuals using two experience questionnaires, one for each of the two races. The scores of the 5 items for each race were averaged for the final scores. For the Caucasian and East Asian experience questionnaires, Cronbach’s $\alpha$ was 0.874 and 0.896, respectively.

### Results

#### Descriptive statistics on implicit social bias and Experience

Table 1 shows the descriptive statistics on the implicit social bias and experience scores. There was a range in implicit social bias toward East Asians. Comparing with the no bias baseline (D score = 0), there was a significant negative bias toward East Asians at the group level, *t*_53_ = − 6.09, *p* < 0.001, Cohen’s *d* = − 0.829 (two-tailed test). The participants also showed a range of experience with East Asian individuals. Overall, participants had less experience with East Asian than Caucasian individuals, *t*_53_ = 4.40, *p* < 0.001, Cohen’s *d* = 0.599.

**Table 1:** Descriptive statistics on implicit social bias and experiences for Experiment 1

	Mean	SD	Max	Min
Implicit social bias toward East Asians	− 0.269	0.325	0.411	− 0.763
Experience with Caucasian individuals	4.24	0.716	5.00	1.80
Experience with East Asian individuals	3.61	0.921	5.00	1.40

#### Group-level performance in the composite task for own- and other-race faces

We first compared the group-level performance in the composite task for Caucasian and East Asian faces (Table 2). A two-way ANOVA was conducted separately on accuracy and correct RT, each with two within-subjects factors: face race (Caucasian vs. East Asian) and congruency (congruent vs. incongruent). The main effect of face race was significant in both accuracy, *F*_1,53_ = 214.93, *p* < 0.001, ${\eta }_{p}^{2}$=0.802, and RT, *F*_1,53_ = 13.53, *p* < 0.001, ${\eta }_{p}^{2}$=0.203, with better and faster performance for Caucasian than East Asian faces, indicating an other-race effect. The main effect of congruency was also found in both accuracy, *F*_1,53_ = 115.27, *p* < 0.001, ${\eta }_{p}^{2}$=0.685, and RT, *F*_1,53_ = 46.34, *p* < 0.001, ${\eta }_{p}^{2}$=0.466, with better and faster performance for the congruent than incongruent trials, revealing holistic processing. The interaction between face race and congruency was not significant in RT, *F*_1,53_ = 2.14, *p* = 0.15, ${\eta }_{p}^{2}$=0.039, but it was significant in accuracy, *F*_1,53_ = 35.41, *p* < 0.001, ${\eta }_{p}^{2}$=0.40, with a larger congruency effect for East Asian than Caucasian faces. Although the larger congruency effect for East Asian than Caucasian faces was unexpected, the congruency effect in accuracy was significant for either face race, as confirmed by one-way ANOVAs with the factor congruency: East Asian faces, *F*_1,53_ = 115.16, *p* < 0.001,${\eta }_{p}^{2}$=0.685; Caucasian faces, *F*_1,53_ = 44.35, *p* < 0.001,${\eta }_{p}^{2}$=0.456. We speculate that the overall reduced magnitude of the congruency effect for own- than other-race faces might be due to the small number of faces used in the current study, which was used to improve reliability of this task for measuring individual differences (Ross et al., 2015), as learning of the small number of specific faces might be easier for own- than other-race faces. Indeed, in a control study, when using a larger number of faces from the same face databases (20 top halves and 20 bottom halves from 40 faces) in a separate group of Caucasian participants (*N* = 36), a two-way ANOVA with face race (Caucasian vs. East Asian) and congruency (congruent vs. incongruent) revealed only a significant main effect of congruency in both accuracy, *F*_1,35_ = 41.11, *p* < 0.001 and RT, *F*_1,35_ = 120.6, *p* < 0.001, a significant main effect of face race in RT, *F*_1,35_ = 21.14, *p* < 0.001 but not in accuracy, *F*_1,35_ = 0.38, *p* = 0.54. More importantly, there was no significant difference between the magnitude of the congruency effects between Caucasian and East Asian faces in either accuracy, *F*_1,35_ = 0.13, *p* = 0.72, or RT, *F*_1,35_ = 0.44, *p* = 0.52. These results of similar holistic processing own- and other-race faces were consistent with previous findings (e.g., Horry et al., 2015; Wong et al., 2021), and thus, the larger congruency effects observed at the group level for East Asian than Caucasian faces in Experiment 1 could likely be influenced by the small number of face stimuli used.

**Table 2:** Group-level performance in the face composite task in Experiment 1

	Accuracy		RT (msec)	
	Congruent	Incongruent	Congruent	Incongruent
East Asian Faces	0.85(0.08)	0.73(0.09)	715 (127)	750(145)
Caucasian Faces	0.92(0.07)	0.87(0.07)	704 (134)	731(141)

Accuracy and correct RT are reported across face race and congruency. Standard errors are reported in parentheses

#### Individual differences analysis: regression analysis on holistic processing of other-race faces

To examine how the magnitude of holistic processing of Caucasian faces, experience with East Asian individuals, and implicit social bias toward East Asians might predict the magnitude of holistic processing of East Asian faces, we conducted multiple regression analyses with the three predictors. The regression analyses were conducted separately on the magnitude of holistic processing measured in accuracy (∆accuracy) and correct RT (∆RT). All predictors were mean-centered.

Table 3 reports the correlations among the dependent measures and the predictors. Table 4 presents the regression results for ∆accuracy. The model with the three predictors (Model 1) explained significant variance in ∆accuracy, *R*^*2*^ = 0.243, *p* = 0.003, *adjusted R*^2^ = 0.198. Holistic processing for Caucasian faces predicted the holistic processing for East Asian faces, $\beta$=0.459, *p* < 0.001, suggesting that participants who process own-race faces more holistically than others also process other-race faces more holistically than others. Importantly, the effect of implicit bias was also significant, $\beta$=0.296, *p* = 0.022. Specifically, participants with positive bias toward East Asian individuals showed stronger holistic processing for East Asian faces than others. The main effect of experience was not significant, $\beta$=− 0.097, *p* = 0.440.

**Table 3:** Correlations among the dependent measures and predictors in Experiment 1

	1	2	3	4	5	6
1. Holistic processing of East Asian faces (∆Accuracy-EA)	–					
2. Holistic processing of East Asian faces (∆RT-EA)	0.130	–				
3. Holistic processing of Caucasian faces (∆Accuracy-C)	0.393**	0.237	–			
4. Holistic processing of Caucasian faces (∆RT-C)	0.203	0.338*	0.066	–		
5. Implicit bias toward East Asians	0.205	0.035	− 0.183	0.182	–	
6. Experience with East Asian individuals	− 0.022	0.205	0.113	0.278*	0.078	–

^*^p < 0.05, **p < 0.005

**Table 4:** Results of the multiple regression analyses on the congruency effect (∆accuracy: accuracy in congruent trials minus accuracy in incongruent trials) in Experiment 1

	Model 1					Model 2				
	$\beta$	*B*	*SE*	*t*	*p*	$\beta$	*B*	*SE*	*t*	*p*
*Predictors*										
Holistic processing of Caucasian faces	0.459	0.624	0.172	3.632	< 0.001	0.393	0.534	0.172	3.114	0.003
Implicit bias toward East Asians	0.296	0.077	0.033	2.356	0.022	0.240	0.062	0.032	1.93	0.060
Experience with East Asians	− 0.097	− 0.01	0.011	− 0.78	0.440	− 0.121	− 0.01	0.011	− 1.00	0.324
*Interaction*										
3-way interaction						− 0.265	− 1.58	0.749	− 2.11	0.040

Model 1 included the three predictors. Model 2 included the three predictors and the significant interaction

Next, we conducted exploratory multiple regression analyses to examine potential interactions among the predictors, given that it is unclear whether and how the three predictors might interact to influence holistic processing of other-race faces. Table 4 (Model 2) presents results from the model with the three-way interaction term. This model explained a larger proportion of variance in ∆accuracy compared with Model 1, *∆R*^2^ = 0.063, *p* = 0.04.^1^ The overall *R*^2^ was also significant, *R*^2^ = 0.306, *p* = 0.001, *adjusted R*^2^ = 0.249. In this model, the effect of holistic processing of Caucasian faces remained significant, $\beta$=0.393, *p* = 0.003, and the effect of implicit social bias approached significance, $\beta$=0.240, *p* = 0.060. There was a significant three-way interaction of holistic processing of Caucasian faces, implicit bias toward East Asians, and experience with East Asians, $\beta$=− 0.265, *p* = 0.040.

**Fig. 3:**
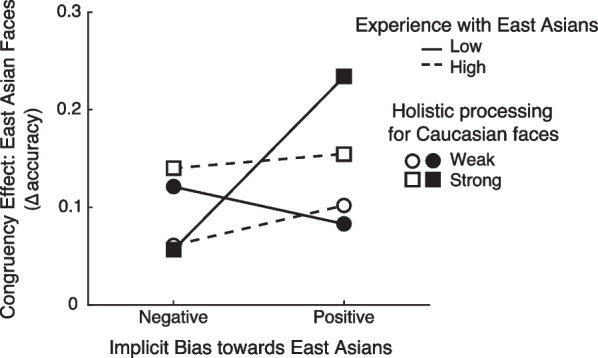
Mean congruency effect for East Asian faces (∆accuracy), as a function of 1*SD* above and below the means of the measures of the magnitude of holistic processing of Caucasian faces (strong vs. weak), experience with East Asian individuals (high vs. low), and implicit bias toward East Asians (positive vs. negative)

The significant three-way interaction is illustrated in Fig. 3. To examine the three-way interaction, simple-slope analyses were conducted. Specifically, different result patterns were observed for the Caucasian participants with high vs. low levels of experience with East Asian individuals. For participants with a lower level of experience with East Asian individuals, those who had strong holistic processing for Caucasian faces showed an effect of implicit bias, with positive, compared with negative, implicit biases toward East Asian individuals predicted an increased magnitude of holistic processing for East Asian faces (*b* = 0.273, *p* < 0.001), but those who had weak holistic processing for Caucasian faces did not show a significant effect of implicit bias (*b* = − 0.059, *p* = 0.45). In contrast, for participants with a higher level of experience with East Asian individuals, only the magnitude of holistic processing for Caucasian faces predicted the magnitude of holistic processing for East Asian faces, and the effect of implicit bias was not significant for either participants who showed strong or weak holistic processing for own-race faces (strong: *b* = 0.023, *p* = 0.80; weak: *b* = 0.063, *p* = 0.30).

For ∆RT, the regression results with the three factors are reported in Table 5. Holistic processing of Caucasian faces predicted holistic processing of East Asian faces, $\beta$=0.309, *p* = 0.031. However, this was not the case for implicit bias: $\beta$= − 0.031, *p* = 0.819, or experience: $\beta$=0.121, *p* = 0.382. Given that holistic processing of Caucasian faces was the only significant predictor in the model, the total variance explained did not reach statistical significance, *F*_3,50_ = 2.45, *p* = 0.074, *adjusted R*^2^ = 0.0759. Potential interactions were also evaluation, and the results showed that none of the interaction effects were significant, |$\beta$|< 0.24, *p* > 0.136.

**Table 5:** Results of the multiple regression analyses on the congruency effect (∆RT: RT in incongruent trials minus RT in congruent trials) in Experiment 1

Predictors	$\beta$	*B*	*SE*	*t*	*p*
Holistic processing of Caucasian faces	0.309	0.307	0.138	2.219	0.031
Implicit bias toward East Asians	− 0.031	− 3.886	16.905	− 0.230	0.819
Experience with East Asians	0.121	5.381	6.101	0.882	0.382

### Discussion

Experiment 1 reveals that holistic processing of other-race faces could be influenced by several factors, including holistic processing of own-race faces and implicit social bias toward other-race group. It is reasonable that the magnitude of holistic processing of own-race faces positively predicted the magnitude of other-race faces and any differences in the processing of own-race and other-race faces are likely quantitative instead of qualitative (DeGutis et al., 2013; Harrison et al., 2014; Horry et al., 2015; Wong et al., 2021), since faces across different races share the same overall configuration (e.g., two eyes above a nose and a mouth).

Importantly, our findings suggest that implicit social bias also had an impact on holistic processing of other-race faces. It is possible that positive bias toward individuals from a different race is associated with positive mood, which is shown to increase holistic processing of faces compared with negative mood (Curby et al., 2012; Xie & Zhang, 2016; Chen & Cheung, 2021). In the exploratory analysis, the three-way interaction of implicit social bias, holistic processing of Caucasian faces, and experience suggests that the effect of implicit social bias on holistic processing of East Asian faces was more pronounced for Caucasian participants with low levels of experience with East Asians than those with high levels of experience with East Asians. Among the participants with low levels of experience with East Asians, those with strong ability to process Caucasian faces may have incorporated social information such as implicit racial bias when processing East Asian faces, with stronger holistic processing for East Asian faces observed in those with more positive implicit social bias toward East Asians. Comparatively the participants with low levels of experience and weak ability to process Caucasian faces were less likely to be influenced by implicit social bias when processing East Asian faces; presumably they might not have the ability to process additional information from the faces. In contrast, for Caucasian participants with high levels of experience with East Asians, the magnitude of holistic processing of East Asian faces was only predicted by the participants’ ability to holistically process Caucasian faces.

To further examine whether these factors similarly influence the processing of other out-group faces, especially the role of implicit social bias, we focused on holistic processing of other-age faces by testing young adult participants on baby faces in Experiment 2. The use of baby faces, instead of elderly faces, was due to the wider range of implicit social biases toward babies than elderly people. Even among young adults who might have relatively limited experience interacting with babies, a wide range of positive to negative implicit social biases toward babies has been observed (Senese et al., 2013). In contrast, the implicit social bias toward elderly people is generally negative (He et al., 2011; Hummert et al., 2002; see also Ebner, 2008; Gluth et al., 2010; Kite et al., 2005). Since the interaction results in Experiment 1 suggest that the influence of implicit social bias on holistic processing was mainly observed in participants with limited experience with individuals from the out-group, Experiment 2 tested young adult participants who had a relatively low level of experience toward babies regarding how implicit social bias toward babies might account for the holistic processing of baby faces, with the two other factors, magnitude of holistic processing of young adult faces and experience toward babies, also taken into account. Similar to Experiment 1, we also explored the potential interactions among the predictors.

## Experiment 2

### Method

#### Participants

A separate group of 62 undergraduate students (32 females and 30 males, mean age = 20.2 years, SD = 1.12) at New York University Abu Dhabi completed Experiment 2 for course credits or subsistence allowance. All participants reported normal or corrected-to-normal vision. They provided written informed consent approved by the New York University Abu Dhabi Institutional Review Board. Using the same exclusion criterion from Experiment 1, three participants were excluded due to low performance in the face composite task (mean accuracy below 65%). The final sample consisted of data from 59 participants.

#### Stimuli

##### Face composite task and IAT

**Fig. 4:**
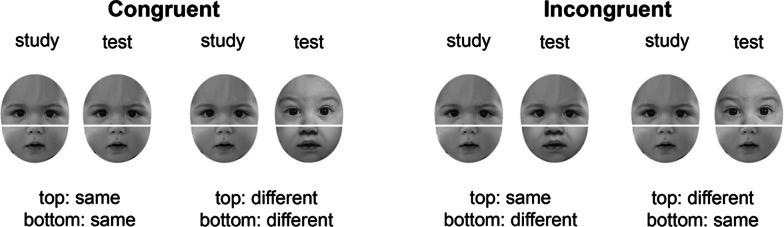
Sample baby stimuli in the face composite task (not included in the actual experiment). The face composites were always aligned. The relationship between the top and bottom halves of the study and test faces could be either congruent or incongruent

Figure 4 illustrates sample stimuli in Experiment 2. The experiment was identical to Experiment 1 except that Caucasian baby faces (under 2 years of age) replaced East Asian faces in both the face composite task and the IAT. The baby faces were obtained from the Internet. Ten baby faces were used in the composite task with five top halves and five bottom halves were selected. Six baby faces were used in the IAT. All baby faces were shown in frontal view.

##### Experience questionnaire

For the experience questionnaires, Experiment 2 adapted the social contact scale from Walker and Hewstone (2006), because the items in the individuating scale used in Experiment 1, also from Walker and Hewstone (2006), were not as appropriate as the social contact scale to describe interactions with babies. The social contact questionnaires included items regarding spending time with from individuals of either of the age groups: babies or young adults. For the questionnaires about babies, the items were ‘How many infants/toddlers (0–3 years old) do you know every well,’ ‘I often spend time with infants/toddlers (0–3 years old),’ ‘I spend a lot of my free time doing things with infants/toddlers (0–3 years old),’ ‘I often go around to the house of infants/toddlers (0–3 years old),’ and ‘Infants/toddlers (0–3 years old) often come around my house.’ Apart from the social contact questionnaire, two additional items were also included: ‘In your day-to-day life, how frequently you see infants/toddlers (0–3 years old),’ ‘In your day-to-day life, how frequently you interact with infants/toddlers (0–3 years old).’ For the questionnaire about young adults, the items used ‘young adults (18–25 years old)’ instead. All the seven items used a 5-point scale. The scores were averaged across the items for each age group, with a high score indicating extensive interactions.

#### Procedure

##### Face composite task, IAT, and experience questionnaires

In the face composite task, participants matched the top halves of either adult or baby composite faces. In the IAT, participants categorized adult vs. baby faces and positive vs. negative words. After completing the face composite task and the IAT, participants completed the experience questionnaires.

The reliability of the congruent trials and the incongruent trials was high for both in-group and out-group faces in both accuracy (*Guttman’s* $\lambda$_*2*_’s > 0.806) and RT (*Guttman’s* $\lambda$_*2*_’s > 0.953) in all conditions. The correlations between the congruent and incongruent trials were also high for accuracy (*r*’s > 0.526) and extremely high in RT (*r*’s > 0.957). The reliability of the difference scores between congruent and incongruent trials for own-age faces were 0.278 (∆accuracy) and 0.113 (∆RT) and that for other-age faces was 0.640 (∆accuracy) and 0.329 (∆RT).

For the reliability of the age IAT in the current study, Cronbach’s $\alpha$ was 0.72 and the correlations between the practice versus test trials were *r* = 0.832, *p* < 0.0001 in the young adult-positive/baby-negative block, and *r* = 0.887, *p* < 0.001 in the young adult-negative/baby-positive block. For the young adult and baby experience questionnaires, Cronbach’s $\alpha$ were 0.607 and 0.836, respectively.

### Results

#### Descriptive statistics on implicit social bias and experience

Table 6 shows the descriptive statistics on the implicit social bias and experience scores. Although the overall implicit bias toward babies was near neutral, since the average D score was not significantly different from the no bias baseline (D score = 0), *t*_58_ = 1.50, *p* = 0.139, Cohen’s *d* = 0.195, the implicit bias scores among individual participants ranged from highly positive vs. highly negative toward babies. The participants had relatively little experience with babies and had significantly more experience with young adults than babies, *t*_58_ = 26.5, *p* < 0.001, Cohen’s *d* = 3.45.

**Table 6:** Descriptive statistics on implicit social bias and experiences for Experiment 2

	Mean	SD	Max	Min
Implicit social bias toward babies	0.061	0.314	0.763	− 0.675
Experience with young adults	4.69	0.340	5.00	3.71
Experience with babies	1.76	0.664	3.43	1.00

#### Group-level performance in the composite task for own- and other-age faces

Table 7 presents the group-level performance in the composite task for adult and baby faces. A two-way ANOVA was conducted separately on accuracy and correct RT, with two within-subjects factors, face age (adult vs. baby) and congruency (congruent vs. incongruent). The main effect of Face Age was significant in both accuracy, *F*_1,58_ = 140.44, *p* < 0.001, ${\eta }_{p}^{2}$=0.708, and RT, *F*_1,58_ = 15.01, *p* < 0.001, ${\eta }_{p}^{2}$=0.206, revealing better and faster performance for adult than baby faces, indicating an other-age effect. The main effect of congruency was significant in both accuracy, *F*_1,58_ = 134.40, *p* < 0.001, ${\eta }_{p}^{2}$=0.699, and RT, *F*_1,58_ = 81.22, *p* < 0.001, ${\eta }_{p}^{2}$=0.583, with better and faster performance for congruent than incongruent trials, revealing holistic processing. The interaction of face age and congruency was significant in both accuracy, *F*_1,58_ = 36.38, *p* < 0.001, ${\eta }_{p}^{2}$=0.385, and RT, *F*_1,58_ = 7.71, *p* = 0.007, ${\eta }_{p}^{2}$=0.117, with a larger congruency effect for baby than adult faces. Similar to the group-level results in Experiment 1, the larger congruency effect for baby than adult faces was not expected and might be due to the use of a small number of faces. Nonetheless, significant congruency effects were observed in both accuracy and RT for both baby faces (accuracy: *F*_1,58_ = 105.55, *p* < 0.001,${\eta }_{p}^{2}$=0.645, RT: *F*_1,58_ = 60.92, *p* < 0.001,${\eta }_{p}^{2}$=0.512) and adult faces (accuracy: *F*_1,58_ = 66.12, *p* < 0.001,${\eta }_{p}^{2}$=0.533, RT: *F*_1,58_ = 28.72, *p* < 0.001,${\eta }_{p}^{2}$=0.331).

**Table 7:** Group-level performance in the face composite task in Experiment 2

	Accuracy		RT (msec)	
	Congruent	Incongruent	Congruent	Incongruent
Adult Faces	0.91 (0.07)	0.85 (.08)	686 (142)	711 (152)
Baby Faces	0.84 (0.10)	0.70 (.11)	695 (140)	740 (145)

Accuracy and correct RT are reported across face age and congruency. Standard errors are reported in parentheses

#### Individual differences analysis: regression analysis on holistic processing for other-age faces

To examine the influences of holistic processing of adult faces, experience with babies, and implicit social bias toward babies on holistic processing of baby faces, we conducted multiple regression analyses with the three predictors on the magnitude of holistic processing of baby faces, measured by ∆accuracy and ∆RT. All predictors were mean-centered.

Table 8 reports the correlations among the dependent measures and the predictors. Table 9 presents the results for ∆accuracy. In Model 1 with the three predictors, only the magnitude of holistic processing of adult faces positively predicted the magnitude of holistic processing of baby faces, $\beta$=0.306, *p* = 0.022. Because the two other factors, implicit social bias toward babies, $\beta$=− 0.761, *p* = 0.559, and experience with babies, $\beta$=− 0.126, *p* = 0.341, were not significant, the total variance explained did not reach statistical significance, *R*^2^ = 0.0995, *F*_3,55_ = 2.02, *p* = 0.121, *adjusted R*^2^ = 0.0503. Further analyses on the interaction effects were conducted, but none of the interaction effects reached significance, |$\beta$|< 0.189, *p* > 0.187.

**Table 8:** Correlations among the dependent measures and predictors in Experiment 2

	1	2	3	4	5	6
1. Holistic processing of baby faces (∆Accuracy-B)	–					
2. Holistic processing of baby faces (∆RT-B)	0.086	–				
3. Holistic processing of young adult faces (∆Accuracy-YA)	0.284*	0.092	–			
4. Holistic processing of young adult faces (∆RT-YA)	0.138	0.098	0.132	–		
5. Implicit bias toward babies	0.051	0.259*	0.026	0.025	–	
6. Experience with babies	− 0.068	0.049	0.156	− 0.162	− 0.133	–

^*^p < 0.05, **p < 0.005

**Table 9:** Results of the multiple regression analysis on the congruency effect (∆accuracy: accuracy in congruent trials minus accuracy in incongruent trials) in Experiment 2

Predictors	$\beta$	*B*	*SE*	*t*	*p*
Holistic processing of young adult faces	0.306	0.578	0.245	2.359	0.022
Implicit bias toward babies	− 0.761	− 0.025	0.042	− 0.59	0.559
Experience with babies	− 0.126	− 0.020	0.020	− 0.96	0.341

Table 10 presents the results for ∆RT. In Model 1 with the three main predictors, the effect of Implicit bias was significant, $\beta$=0.270, *p* = 0.042, suggesting that participants with positive bias toward babies showed stronger holistic processing for baby faces than participants with negative bias toward babies. However, both holistic processing of adult faces, $\beta$=0.108, *p* = 0.414 and experience with babies, $\beta$=0.102, *p* = 0.441, were not significant. Thus, the model with the three predictors did not significantly explain the total variance, *R*^2^ = 0.293, *F*_3,55_ = 1.72, *p* = 0.174, *adjusted R*^2^ = 0.0358. More importantly, Table 10 also presents the results of Model 2 with the three predictors and the 2-way interaction of the magnitude of holistic processing of adult faces and implicit social bias. The effect of implicit social bias remained significant, $\beta$=0.295, *p* = 0.023, with implicit social bias positively predicted the magnitude of holistic processing of baby faces. The interaction between holistic processing of adult faces and Implicit social bias was also significant, $\beta$=0.284, *p* = 0.030. The overall R^2^ was significant for Model 2, *R*^2^ = 0.1625, *p* = 0.045, *adjusted R*^2^ = 0.10,^2^ and Model 2 explained a larger proportion of variance in ∆RT compared with Model 1, *∆R*^2^ = 0.0768, *p* = 0.03.

**Table 10:** Results of the multiple regression analyses on the congruency effect (∆RT: RT in incongruent trials minus RT in congruent trials) in Experiment 2

	Model 1					Model 2				
	$\beta$	*B*	*SE*	*t*	*p*	$\beta$	*B*	*SE*	*t*	*p*
*Predictors*										
Holistic processing of adult faces	0.108	0.131	0.159	0.823	0.414	0.163	0.199	0.157	1.270	0.210
Implicit bias toward babies	0.270	37.987	18.280	2.078	0.042	0.295	41.421	17.723	2.337	0.023
Experience with babies	0.102	6.807	8.766	0.777	0.441	0.101	6.743	8.467	0.796	0.429
*Interaction*										
Holistic processing of adult faces $\times$ Implicit bias						0.284	1.069	0.480	2.226	0.030

Model 1 included the three main factors. Model 2 included the three main factors and the significant interaction

**Fig. 5:**
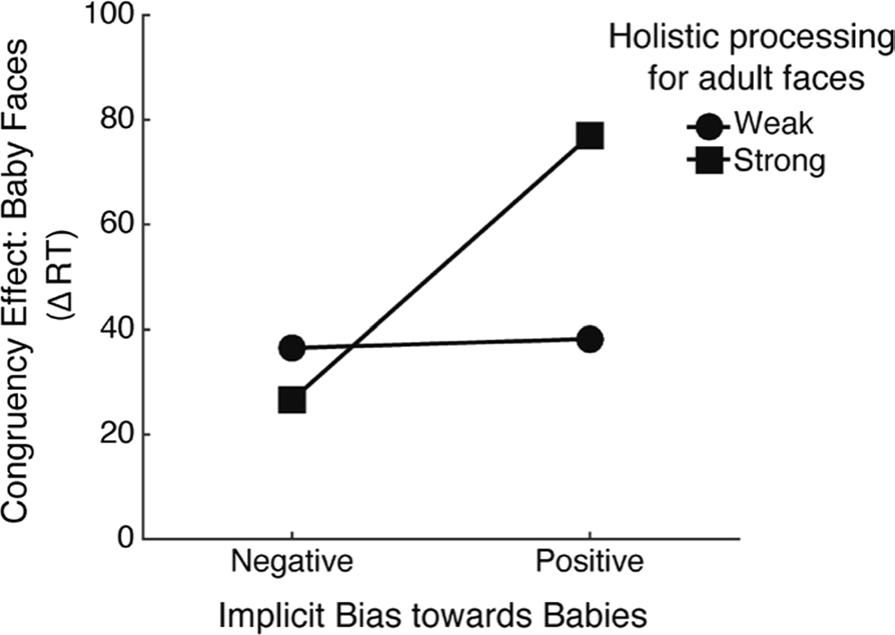
Mean congruency effect for baby faces (∆RT), as a function of 1*SD* above and below the means of the magnitude of holistic processing of adult faces (strong vs. weak) and implicit bias toward babies (positive vs. negative)

Figure 5 illustrates the two-way interaction revealed in Model 2 between Holistic processing of adult faces and implicit social bias in ∆RT. Simple-slope analyses showed that for these participants who had relatively limited experience with babies, positive implicit bias resulted in increased holistic processing of baby faces among those with strong holistic processing of adult faces in participants (*b* = 80.10, *t* = 3.10, *p* = 0.003), but not among those with weak holistic processing of adult faces (*b* = 2.742, *t* = 0.12, *p* = 0.91).

### Discussion

Experiment 2 replicated the main findings of Experiment 1 regarding the influence of holistic processing of in-group faces and implicit social bias toward the out-group on holistic processing of out-group faces. We found that the magnitude of holistic processing of young adult faces positively predicted the magnitude of holistic processing of baby faces across individual participants. Social experience with babies did not appear to predict the magnitude of holistic processing of baby faces, presumably because most of the participants in Experiment 2 had relatively little experience with babies. More importantly, the significant interaction between implicit social bias toward babies and holistic processing for young adult faces suggested that the influence of implicit social bias on holistic processing of baby faces was predominantly observed in participants with strong holistic processing for young adult faces, but not in participants with weak holistic processing for young adult faces, suggesting that implicit social bias may not be utilized by all participants, but more likely by those who have superior holistic processing ability to flexibly utilize various kinds of information.

Because all participants in Experiment 2 had relatively limited experience with babies, the significant interaction between holistic processing of own-age group and implicit social bias toward other-age group was consistent with the interaction results observed in Experiment 1 that the effect of implicit social bias on holistic processing of out-group faces was mainly found in participants with high holistic processing ability for in-group faces and low levels of experience with the out-group.

## General discussion

The current study investigated how three factors: implicit social bias toward the out-group, experience with members of the out-group, and holistic processing ability for in-group faces might predict holistic processing of other-group faces. Note that although not all predictors were significantly correlated with holistic processing of out-group faces, all predictors were included in the regression analyses because they were thought to influence holistic processing of out-group faces, based on previous research. The interactions among the predictors further suggested that the effect of implicit social bias depended on the levels of other predictors, revealing moderation effects (Hayes, 2014).

Replicating previous findings (e.g., Horry et al., 2015), holistic processing of in-group faces significantly predicted holistic processing of out-group faces, suggesting similar processes for the different groups of faces. More importantly, across two experiments, there was consistent evidence for the role of implicit social bias on holistic processing of both other-race and other-age faces. Specifically, the interactions of the factors suggested that the effect of implicit social bias depends on additional factors such as contact experience and holistic processing ability. Indeed, the effect of implicit social bias was strongest in observers who demonstrated strong holistic processing ability for in-group faces who had limited contact experience with the out-group members.

Consistent with the notion that any differences between holistic processing for the different groups are quantitative instead of qualitative (DeGutis et al., 2013; Harrison et al., 2014; Horry et al., 2015; Wong et al., 2021), we found that the magnitudes of holistic processing for in-group and out-group faces were correlated in both experiments. Because there is a wide range of individual differences in face recognition and/or holistic processing ability (DeGutis et al., 2013; Richler et al., 2011; Wang et al., 2012), it is conceivable that these differences could influence whether different kinds of perceptual or socio-cognitive information may be used during face processing. Extending from previous findings, the interaction results suggest that the observer’s holistic processing ability may also determine whether other aspects of facial information that may be derived from social evaluation, such as implicit social bias, are also processed for out-group faces. It is possible that while observers with weak holistic processing ability might struggle to process all aspects of facial information, those with strong holistic ability instead have the capacity to not only process physical facial features and their configurations as a whole, but also social features such as race and age categorization (Levin, 1996; 2000).

Previous studies have shown that increased contact experience in individuating out-group faces not only enhances holistic processing of other-race or other-age faces and improves recognition performance (e.g., Bukach et al., 2012), but also reduces negative implicit bias toward the out-group (Lebrecht et al., 2009; Walker & Hewstone, 2008). Because the effect of implicit social bias on holistic processing of out-group faces was more likely found in observers who had limited experience with members of the out-groups, it is possible that having extensive experience with members of other race or age groups is sufficient to efficiently process out-group faces, and thus, additional information from social evaluation is not utilized. In contrast, positive social evaluation information, similar to the effect of positive emotions (Chen & Cheung, 2021; Curby et al., 2012; Xie & Zhang, 2016), may prompt observers who have little experience with the out-groups to further engage holistic processing when recognizing those faces. Importantly, note that experience and implicit social bias do not necessarily account for the same variance in the processing of out-group faces (Trawiński et al., 2021). In particular, we observed no significant correlations between experience with an out-group and implicit social bias toward the specific group in either experiment. Instead, implicit social bias and experience could have separate contributions to the holistic processing of out-group faces.

The current findings suggest that implicit social bias might have different impact among observers. Given the potential interactions of holistic processing ability, experience with members of the out-group, and implicit social bias toward the out-group, it may be unsurprising that previous studies have reported mixed results in socio-cognitive influences on the processing of other-race faces (e.g., Hugenberg et al., 2010; Kloth et al., 2014; Wan et al., 2015). Instead, the influence of socio-cognitive factors might be particularly robust and consistent in observers who have little experience with the out-groups and strong holistic processing ability.

Race and age are salient but vastly different visual information for categorizing faces. While the other-race effect has been very well documented, relatively little research has been conducted on the other-age effect, and only a handful of studies reported both effects in the same study (e.g., Wiese et al., 2013). Most researchers have assumed that the other-race and other-age effects share similar mechanisms (e.g., Rhodes & Anastasi, 2012; but see Wiese et al., 2013). In the current study, to illustrate the potential influence of implicit social bias on holistic processing of out-group faces, Caucasian participants completed the tasks on Caucasian and East Asian faces (Experiment 1) and young adult participants completed the tasks on young adult and baby faces (Experiment 2). Although it might be ideal to also examine these effects in participants from other race or age groups (e.g., Black/African faces, elderly faces), there were practical concerns regarding whether it would be possible to acquire a sufficient range of variability in the implicit social biases toward the out-groups. We were able to obtain a range of positive to negative implicit biases toward East Asians and babies in our participants. However, it remains highly challenging to obtain such variability in other race or age groups. For instance, there are generally positive implicit biases toward Caucasians and young adults, and negative implicit biases toward Black/Africans and elderly adults. These constraints make it difficult to demonstrate a reversed pattern of results from East Asian participants with Caucasian faces. Nonetheless, because the main findings were consistent across the other-race group in Experiment 1 and the other-age group in Experiment 2, it is likely that the influence of implicit social bias on holistic processing of faces from different out-groups is reliable.

The current study has expanded a growing literature on the influence of implicit social bias on face recognition (Lebrecht et al., 2009; Walker & Hewstone, 2008; Trawiński et al., 2021) by demonstrating its effect on holistic processing of other-race and other-age faces. It is important to note, however, that even though holistic processing is critical for face recognition, other types of processing, such as featural processing of individual components on a face (e.g., when only the eyes are available), also contribute to successful face recognition. Indeed, both holistic and featural processing could be impaired for out-group, compared with in-group, faces (Hayward et al., 2013). Future studies should examine whether other crucial aspects of face recognition, such as featural processing, might also be similarly influenced by various factors such as implicit social bias toward the out-group and social experience with members of out-group. Here, we provided a framework to study these factors in understanding the perceptual and socio-cognitive influences on recognition of out-group faces.

## Data Availability

The dataset and analysis code are available on https://osf.io/dkj8r/.

## Abbreviation

IAT: Implicit association test

## Footnotes

1 The variance inflation factor (VIF) estimates suggested that there were no multicollinearity issues among any of the three factors and their interactions (VIF_HP_ACC_Caucasian_ = 1.12; VIF_IAT_ = 1.10; VIF_Experience_ = 1.03; VIF _IAT Experience HP_ACC_Caucasian_ = 1.12).

2 The variance inflation factor (VIF) estimates suggested that there were no multicollinearity issues among any of the factors and their interactions (VIF_HP_RT_Adult_ = 1.07; VIF_IAT_ = 1.03; VIF_Experience_ = 1.05; VIF_IAT HP_RT_Adult_ = 1.05).
